# The Impact of Coexistent Hashimoto’s Thyroiditis on Central Compartment Lymph Node Metastasis in Papillary Thyroid Carcinoma

**DOI:** 10.3389/fendo.2021.772071

**Published:** 2021-11-16

**Authors:** Yang Liu, Hongjun Lv, Shaoqiang Zhang, Bingyin Shi, Yushi Sun

**Affiliations:** ^1^ Department of Breast Surgery, The First Affiliated Hospital of Xi’an Jiaotong University, Xi’an, China; ^2^ Department of Endocrinology, The First Affiliated Hospital of Xi’an Jiaotong University, Xi’an, China; ^3^ Department of Otolaryngology-Head and Neck Surgery, The First Affiliated Hospital of Xi’an Jiaotong University, Xi’an, China

**Keywords:** papillary thyroid carcinoma, Hashimoto’s thyroiditis, central compartment lymph node metastasis, multivariate analysis, receiver operating characteristic analysis

## Abstract

**Background:**

Hashimoto’s thyroiditis (HT) is the most prevalent inflammatory disorder of the thyroid gland. Current studies have reported the coexistence rate between HT and papillary thyroid carcinoma (PTC) is quite high. The objective of this study was to evaluate the impact of HT on the predictive factors of central compartment lymph node metastasis (CLNM) in PTC.

**Methods:**

A retrospective investigation was performed on PTC patients. They were subclassified into HT and non-HT groups. The results of preoperative neck ultrasound (US) examinations were reviewed. The clinical characteristics and the predictive value for CLNM were explored and compared between the two groups.

**Results:**

A total of 756 patients were included in this study. There were more female patients (86.1%) in the PTC coexistent with the HT group than non-HT group. The patients with HT group had higher preoperative serum level of TSH. There was statistically significant difference between the HT patients and non-HT patients in nodular vascularization. Univariate and multivariate analyses showed that male, age ≤45 years old, tumor diameter >1 cm, and presence of suspicious central compartment lymph node on US, irregular nodular shape, multifocal carcinoma were independent predictive factors of CLNM in PTC patients. It was showed that male, age ≤45 years old, tumor diameter >1 cm, multifocality, and presence of suspicious central lymph node on US were risk factors for CLNM in non-HT patients. Only tumor diameter >1 cm and presence of suspicious central lymph node on US were independently correlated with CLNM in HT patients. The sensitivity of the multivariate model was 63.5%, and specificity was 88.9% for prediction CLNM in HT patients. For non-HT patients, the AUC was 80.6%, the sensitivity of the multivariate model was 64.5%, and specificity was 85.2%

**Conclusion:**

PTC combined with HT is more common in women, and TSH level in HT group is higher than that in patients with PTC alone. Regardless of that HT is not a related risk factor of CLNM in PTC, our result suggested that different predictive systems should be used for HT and non-HT patients respectively to have a more accurate evaluation of CLNM in clinic.

## Introduction

Hashimoto’s thyroiditis (HT), also known as chronic lymphocytic thyroiditis, is the most prevalent inflammatory disorder of the thyroid gland, with an incidence of 0.3%–5.1% (1, 2). Thyroid cancer is the most common malignant tumor of the endocrine system, and its incidence rate is rapidly increasing with an annual growth rate of 4.5%–6.6% (3). The papillary thyroid carcinoma (PTC) accounts for 80%–90% of all thyroid malignant tumors (3, 4).

Since Dailey et al. (5) proposed the association between HT and PTC in 1952, many etiological and epidemiological studies have focused on the relationship between the two diseases. Notably, current studies have reported that the average coexistence rate between HT and PTC is quite high, approximately 23% (range from 10% to 58%) (6). Several previous reports have shown that PTC coexistent with HT is associated with a better prognosis (7–10). However, this conclusion has not been confirmed in some other studies, and the pathogenesis of the coexistence of PTC and HT remains controversial (11, 12).

PTC is usually considered as an indolent tumor and mostly has a good prognosis. However, it is prone to lymph node metastasis, especially central compartment lymph node metastasis (CLNM). In addition, there is still a significant controversy regarding routine prophylactic central compartment lymph node dissection in cN0 PTC patients because of the potential high incidence of postoperative complications and uncertainty of improved prognosis (13, 14). At present, neck ultrasound (US) is the most valuable method for preoperative evaluation of lymph node status (15–17). However, the sensitivity of US for predicting CLNM in PTC is only 23%–30.0% (15, 18).

Recurrent laryngeal nerve (RLN) injury is one of the most common causes of litigation after thyroidectomy (19). RLN paralysis after lymph node dissection can be unilateral or bilateral. It can result in a group of voice symptoms such as breathiness due to air leakage, hoarseness, and vocal fatigue leading to using short sentences in unilateral paralysis of RLN (20). Pereira found that voice changes after thyroidectomy with intact voice nerves were present in 28% of the involved patients (21). Several studies have shown that voice changes after lymph node dissection have a negative impact on life (20). Accurate preoperative evaluation of CLNM is particularly crucial for determining the appropriate scope of lymph node dissection. However, there are few studies on the effects of coexisting HT with PTC on the characteristics of lymph node metastasis in the central region. In this study, PTC patients were subclassified into HT and non-HT groups. The clinical characteristics and the predictive value for CLNM were explored and compared between the two groups.

## Materials and Methods

A retrospective investigation was performed on PTC patients at The First Affiliated Hospital of Xi’an Jiaotong University from January 2014 to May 2017. The study was approved by the institutional review board. All of the PTC patients had received initial thyroidectomy with at least one side central neck dissection (CND). Those who did not receive CND were excluded from this study. Patients who had received preoperative I^131^ ablation or prior head and neck oncological surgery were excluded from our study. Those who had undergone TSH suppression therapy or antithyroid therapy before surgery were also excluded (22). A total of 756 patients were included in this study. The mean age was 42 years (range, 9–84 years). Patients were staged according to the American Joint Committee on Cancer (AJCC) 8th edition of the tumor–node–metastasis (TNM) staging standard for thyroid cancer (23). HT was defined as the presence of diffuse lymphoplasmacytic infiltration, germinal centers, and enlarged epithelial cells with large nuclei and eosinophilic cytoplasm (24). Coexistence of HT with PTC was confirmed by the postoperative pathological examination in 130 (17.20%) patients. CLNM was histologically proven in 60.19% (455/756) patients. Patients were divided into HT group (n = 130) and non-HT group (n = 626). The clinical features of PTC were compared between the two groups.

The measurements of preoperative serum thyroid function and thyroid relative autoantibodies were done by radioimmunoassay. The results of preoperative neck US examinations were reviewed. The ultrasonographic characteristics of the suspicious thyroid nodules, including size, number (multifocal/unifocal), location (bilateral/unilateral), shape (regular/irregular), border (clear/obscure), echogenicity (hypoechoic/hyperechoic or isoechoic), calcification (non-calcification/microcalcification/coarse calcification), and degree of vascularization (none/low/middle/high), were recorded. Thyroid nodules that were diagnosed TI-RADS fourth or fifth grade by radiologists were defined as suspicious thyroid nodules in this study. The diameter of the largest suspicious thyroid nodule was used as tumor size for analysis. Lymph node showing one or more suspicious features (focal or diffuse hyperechogenicity, presence of internal calcification, cystic change, round shape, or chaotic vascularity) on US was regarded as clinical pathologic lymph nodes (22).

SPSS statistical software version 22.0 (SPSS Inc, Chicago, IL) was used to analyze the data. p < 0.05 was considered statistically significant. Differences in single variables were tested with the chi-square test or unpaired non-parametric test (Mann–Whitney U-test). Multivariate analysis using logistic regression analysis was performed on the variables that showed p < 0.1 in univariate analysis. Predictive value of those factors was measured using the area under the receiver operating characteristic (ROC) curve.

## Results

### Basic Clinical Features of HT Group and Non-HT Group

There were more female patients (86.1%) in the PTC coexistent with the HT group than non-HT group (p < 0.001). Compared with non-HT group, the patients with HT group had higher preoperative serum level of TSH (p < 0.05). There was statistically significant difference between the HT patients and non-HT patients in nodular vascularization (p = 0.022). There were no statistically significant differences between the two groups in age, preoperative T4 level, T3 level, nodular size, number, location, nodular shape, border, internal echo, and calcification, and the presence of suspicious central compartment lymph node (CLN) on US (*p* > 0.05). There was no difference in the CLNM rate between the HT and non-HT groups. During lymphadenectomy, 0–41 lymph nodes were removed in the central compartment region. The number of lymph node dissections in the central compartment region of PTC coexistent with the HT group was more than those in the PTC group (*p <* 0.001; **Table 1**). However, the number of metastatic lymph nodes in the central compartment region was fewer in the HT group (*p* = 0.02; **Table 1**). There were more patients with stage I (94.6%) in HT group than in non-HT group (*p* < 0.001; **Table 1**).

**Table 1:** Clinicopathological characteristic of HT and non-HT PTC patients.

	HT (n = 130)	Non-HT (n = 626)	*p*
Age at diagnosis, M (P_25_; P_75_)	40 (31; 48)	43 (33; 51)	0.107
Age (≤45 y/>45 y)	85/45 65.4%/34.6%	369/257 58.9%/41.1%	0.137
Gender (male/female)	18/112 13.8%/86.2%	181/445 28.9%/71.1%	<0.001
TSH, μIU/ml, M (P_25_; P_75_)	2.42 (1.44; 3.97)	1.96 (1.24; 3.02)	0.013
T4, μg/dl, M (P_25_; P_75_)	15.4 (12.7; 17.1)	15.6 (13.7; 17.5)	0.309
T3, ng/ml, M (P_25_; P_75_)	5.27 (4.49; 6.01)	5.38 (4.67; 6.07)	0.328
**Ultrasonographic characteristics of suspicious nodules**			
Tumor size (≤1 cm/>1 cm)	39/91 30%/70%	151/475 24.1%/75.9%	0.151
Unilateral/bilateral	86/44 66.1%/33.9%	451/175 72%/28%	0.178
Unifocal/multifocal	72/58 55.3%/44.7%	358/268 57.2%/42.8%	0.705
Border (clear/obscure)	66/64 50.7%/49.3%	312/314 49.8%/50.2%	0.847
Margin (regular/irregular)	60/70 46.1%/53.9%	253/373 40.4%/59.6%	0.227
Non-/micro-/coarse calcification	26/92/12 20%/70.7%/9.3%	116/442/88 18.5%/70.6%/10.9%	0.333
Hypoechoic/hyper- or isoechoic	121/9 93.1%/6.9%	605/21 96.6%/3.4%	0.058
Vascularization, none/low/middle/high	49/36/31/14 37.7%/27.7%/23.8%/10.8%	173/188/166/99 27.6%/30%/26.5%15.9%	0.022
Absence/presence of suspicious CLN on ultrasonography	107/23 82.3%/17.7%	474/152 75.7%/24.3%	0.105
**CLNM**			
CLNM, presence/absence	70/60 53.8%/46.2%	385/241 61.5%/38.5%	0.105
Number of removed CLNs, M(P_25_; P_75_)	9 (6; 13)	5 (3; 9)	<0.001
Number of metastatic CLNS, M (P_25_; P_75_)	1 (0; 3)	1 (0; 4)	0.02
TNM staging			<0.001
Stage I	123 (94.6%)	578 (92.3%)	
Stage II	5 (3.9%)	40 (6.4%)	
Stage III	2 (1.5%)	8 (1.3%)	
Stage IV	0 (0)	0 (0)	

CLNM, central lymph node metastases; CLN, central lymph node; PTC, papillary thyroid cancer; M, median value.

### Sonographic Characteristics of Suspicious Lymph Node

A total of 175 patients were regarded as clinical pathological lymph nodes (cN1) in this study. There were 152 patients with round shape (86.8%), 76 with internal calcification (43.4%), 64 with focal or diffuse hyperechogenicity (36.6%), 21 with cystic change (12%), and 14 with chaotic vascularity (8%). There were 23 patients with HT in the cN1 group. Seventeen of the 23 (73.9%) patients were round shape on US. There were no statistically significant differences between the two groups in focal or diffuse hyperechogenicity, presence of internal calcification, cystic change, round shape, and chaotic vascularity on US (p > 0.05; **Table 2**).

**Table 2:** Sonographic characteristics of suspicious lymph node.

	HT (23)	Non-HT (152)	*p*
Round shape (yes/no)	17/6 73.9%/26.1%	135/17 88.8%/11.2%	0.101
Internal calcification (yes/no)	13/10 56.5%/43.5%	63/89 41.4%/58.6%	0.174
Cystic change (yes/no)	3/20 13%/87%	18/134 11.8%/88.2%	1.000
Hyperechogenicity (yes/no)	7/16 30.4%/69.6%	57/95 37.5%/62.5%	0.512
Chaotic vascularity (yes/no)	2/21 8.7%/91.3%	12/140 7.9%/92.1%	1.000

### Predictive Risk Factors of CLNM in PTC Patients

Univariate analysis showed that age, gender, preoperative T4 level, T3 level, nodular size, number, location, nodular shape, border, calcification, presence of suspicious CLN on US, and the number of lymph node dissections in the central compartment region were all correlated with CLNM (*p* < 0.05; **Table 3**). Then, multivariate analysis was used to detect the risk factors of CLNM in PTC patients. It was showed that male, age ≤45 years old, tumor diameter >1 cm, and presence of suspicious CLN on US, irregular nodular shape, and multifocal carcinoma were all risk factors for CLNM in PTC patients (**Table 4**).

**Table 3:** Univariate analysis of the correlation between clinical factors of the primary tumor and rate of CLNM in PTC patients.

	CLNM (n = 455)	NCLNM (n = 301)	*p*
Age at diagnosis, M(P_25_; P_75_)	38 (30; 48)	47 (29; 53)	<0.001
Age (≤45 years/>45 years)	323/132 71.0%/29.0%	131/170 43.5%/56.5%	<0.001
Gender (male/female)	149/306 32.7%/67.3%	50/251 16.6%/83.4%	<0.001
Hashimoto’s thyroiditis, absent/present	385/70 84.6%/15.4%	241/60 80.1%/19.9%	0.105
TSH, μIU/ml, M (P_25_; P_75_)	2.03 (1.28; 3.02)	1.85 (1.27; 3.21)	0.614
T4, μg/dl, M (P_25_; P_75_)	15.7 (13.9; 17.6)	15.3 (13.1; 16.9)	0.018
T3, ng/ml, M(P_25_; P_75_)	5.48 (4.71; 6.15)	5.21 (4.55; 5.98)	0.017
**Ultrasonographic characteristics of suspicious nodules**			
Tumor size (≤1 cm/>1 cm)	79/376 17.4%/82.6%	111/190 36.9%/63.1%	<0.001
Unilateral/bilateral	311/144 68.4%/31.6%	226/75 75.1%/24.9%	0.046
Unifocal/multifocal	236/219 51.9%/48.1%	194/107 64.5%/35.5%	0.001
Border (clear/obscure)	214/241 47.0%/53.0%	164/137 54.5%/45.5%	0.045
Margin (regular/irregular)	165/290 36.3%/63.7%	148/153 49.2%/50.8%	<0.001
Non-/micro-/coarse calcifification	62/336/57 13.6%/73.9%/12.5%	80/178/43 26.6%/59.1%/14.3%	<0.001
Hypoechoic/hyper- or isoechoic	437/18 96.0%/4%	289/12 96.0%/4.0%	0.983
Vascularization None/low/middle/high	134/127/118/76 29.5%/27.9%/25.9%/16.7%	88/97/79/37 29.2%/32.2%/26.3%/12.3%	0.338
absence/presence of suspicious CLN on ultrasonography	295/160 64.8%/35.2%	286/15 95%/5%	<0.001
Number of removed CLNs, M(P_25_; P_75_)	7 (4; 10)	5 (2; 8)	<0.001

CLNM, central lymph node metastases; CLN, central lymph node; PTC, papillary thyroid cancer; M, Median value.

**Table 4:** Multivariate analysis of the correlation between clinical factors of the primary tumor and rate of CLNM in all PTC patients.

Variables	OR	CI	*p*
Gender (male)	2.278	1.458-3.56	<0.001
Age (≤45 years)	3.211	2.222-4.641	<0.001
Margin (regular)	0.629	0.425-0.930	0.020
Multifocal	2.444	1.437-4.159	0.001
Tumor size (≤1 cm)	0.314	0.207-0.476	<0.001
Presence of suspicious CLN on ultrasonography	8.470	4.597-15.606	<0.001

CLNM, central lymph node metastases; CLN, central lymph node; PTC, papillary thyroid cancer.

**Figure 1:**
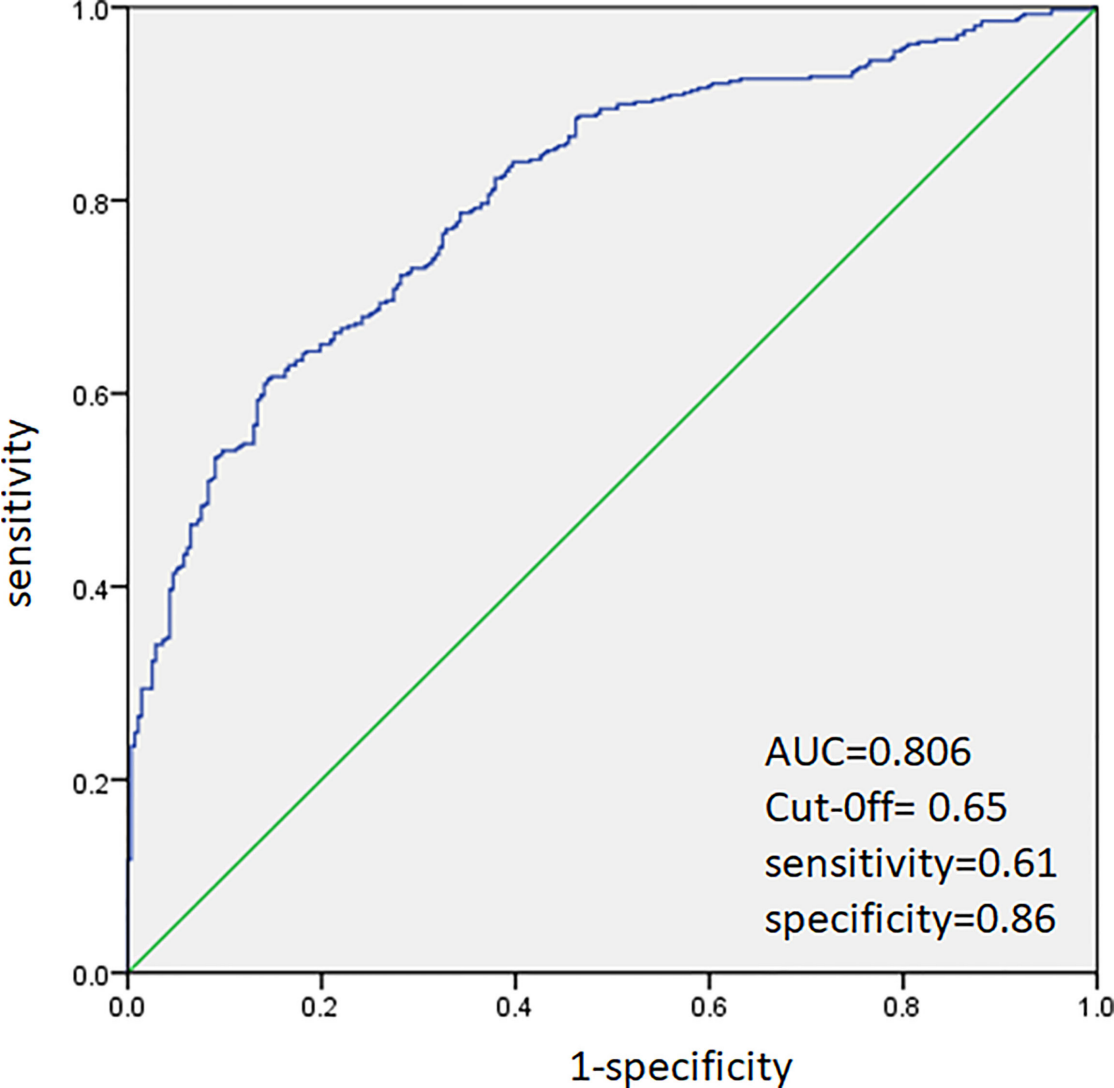
Receiver operating characteristic curve analysis for prediction of central lymph node metastases using the multivariate model in PTC patients.

ROC analysis was performed to predict CLNM in PTC patients. The area under the curve (AUC) was 80.6% (**Figure 1**). A cutoff point for the prediction of CLNM was defined as a value 65% for PTC patients. The sensitivity of the multivariate model was 61%, and specificity was 86% for prediction CLNM in PTC patients (**Figure 1**).

### Predictive Risk Factors of CLNM in HT Patients and Non-HT Patients

Patients were divided into HT group and non-HT group. In univariate analysis, age (*p* = 0.021), gender (*p* = 0.028), T3 (*p* = 0.022), nodular size (*p* = 0.002), nodular number (*p* = 0.041), and presence of suspicious central cervical lymph node on US (*P*<0.001) were all correlated with CLNM in HT patients. In non-HT patients, age (*p* < 0.001), gender (*p* < 0.001), T4 (*p* = 0.035), nodular size (*p* < 0.001), nodular number (*p* = 0.004), nodular shape (*p* = 0.003), calcification (*p* < 0.001) and presence of suspicious central cervical lymph node on US (*p* < 0.001) were significantly associated with CLNM (**Table 5**).

**Table 5:** Univariate analysis of the correlation between clinical factors of the primary tumor and rate of CLNM in HT and non-HT PTC patients.

	HT (n = 130)			Non-HT (n =626)		
	CLNM (n = 70)	NCLNM (n = 60)	*p*	CLNM (n = 385)	NCLNM (n = 241)	*p*
Age at diagnosis, M(P_25_; P_75_)	37 (31; 47)	42.5 (31; 51)	0.034	38 (30; 48)	48 (40; 53)	<0.001
Age (≤45 years/>45 years)	52/18 74.3%/25.7%	33/27 55.0%/45.0%	0.021	271/114 70.4%/29.6%	98/143 40.7%/59.3%	<0.001
Gender (male/female)	14/56 20%/80%	4/56 6.7%/93.3%	0.028	135/250 35.0%/65.0%	46/195 19.1%/80.9%	<0.001
TSH, μIU/ml, M (P_25_; P_75_)	2.33 (1.28;3.72)	2.53 (1.58;4.13)	0.721	2.01 (1.27; 2.90)	1.76 (1.23; 3.14)	0.353
T4, μg/dl, M (P_25_; P_75_)	15.8 (12.6;17.6)	15 (12.7;16.9)	0.419	15.7 (13.9; 17.6)	15.3 (13.5; 16.8)	0.035
T3, ng/ml, M (P_25_; P_75_)	5.61 (4.61;6.41)	4.99 (4.45;5.63)	0.022	5.46 (4.73; 6.07)	5.26 (4.59; 6.1)	0.141
**Ultrasonographic characteristics of suspicious nodules**						
Tumor size (≤1 cm/>1 cm)	13/57 18.6%/81.4%	26/34 43.3%/56.7%	0.002	66/319 17.1%/82.9%	85/156 35.3%/64.7%	<0.001
Unilateral/bilateral	41/29 58.6%/41.4%	45/15 75.0%/25.0%	0.048	270/115 70.1%/29.9%	181/60 75.1%/24.9%	0.177
Unifocal/multifocal	33/37 47.1%/52.9%	39/21 65%/35.0%	0.041	203/182 52.7%/47.3%	155/86 64.3%/35.7%	0.004
Border (clear/obscure)	34/36 48.6%/51.4%	32/28 53.3%/46.7%	0.588	180/205 46.8%/53.2%	132/109 54.8%/45.2%	0.051
Margin (regular/irregular)	27/43 38.6%/61.4%	33/27 55.0%/45.0%	0.061	138/247 35.8%/64.2%	115/126 47.7%/52.3%	0.003
Non-/micro-/coarse calcification	12/51/7 17.1%/72.9%/10%	14/41/5 23.3%/68.3%/8.4%	0.667	50/285/50 13.0%/74.0%/13.0%	66/137/38 27.4%/56.8%/15.8%	<0.001
Hypoechoic/hyper- or isoechoic	64/6 91.4%/8.6%	57/3 95.0%/5.0%	0.504	373/12 96.9%/3.1%	232/9 96.3%/3.7%	0.676
Vascularization None/low/middle/high	30/15/17/8 42.8%/21.4%/24.3%/11.5%	19/21/14/6 31.7%/35.0%/23.3%/10.0%	0.565	104/112/101/68 27.0%/29.1%/26.2%/17.7%	69/76/65/31 28.6%/31.5%/27.0%/12.9%	0.250
Absence/presence of suspicious CLN on ultrasonography	49/21 70.0%/30.0%	58/2 96.7%/3.3%	<0.001	246/139 63.9%/36.1%	228/13 94.6%/5.4%	<0.001

CLNM, central lymph node metastases; CLN, central lymph node; PTC, papillary thyroid cancer; M, Median value.

Next, we investigated the risk factors associated with CLNM in HT patients and non-HT patients. It was showed that male, age ≤45 years old, tumor diameter >1 cm, multifocality, and presence of suspicious CLN on US were all risk factors for CLNM in non-HT patients (**Table 6**). However, only tumor diameter >1 cm and presence of suspicious CLN on US were independently correlated with CLNM in HT patients (**Table 6**).

**Table 6:** Multivariate analysis of the correlation between clinical factors of the primary tumor and rate of CLNM in HT and non-HT patients.

Variables	OR	CI	*p*
**Non-HT group**			
Age (≤45 years)	3.323	2.223–4.968	<0.001
Gender (male)	2.156	1.346–3.454	0.001
Tumor size (≤1 cm)	.378	0.240–0.597	<0.001
Multifocal	1.669	1.109–2.510	0.014
Presence of suspicious CLN on ultrasonography	8.0	4.132–15.488	<0.001
**HT group**			
Tumor size (≤1 cm)	0.181	.057–0.572	0.004
Presence of suspicious CLN on ultrasonography	14.743	2.593–83.821	0.002

CLNM, central lymph node metastases; CLN, central lymph node; PTC, papillary thyroid cancer.

**Figure 2:**
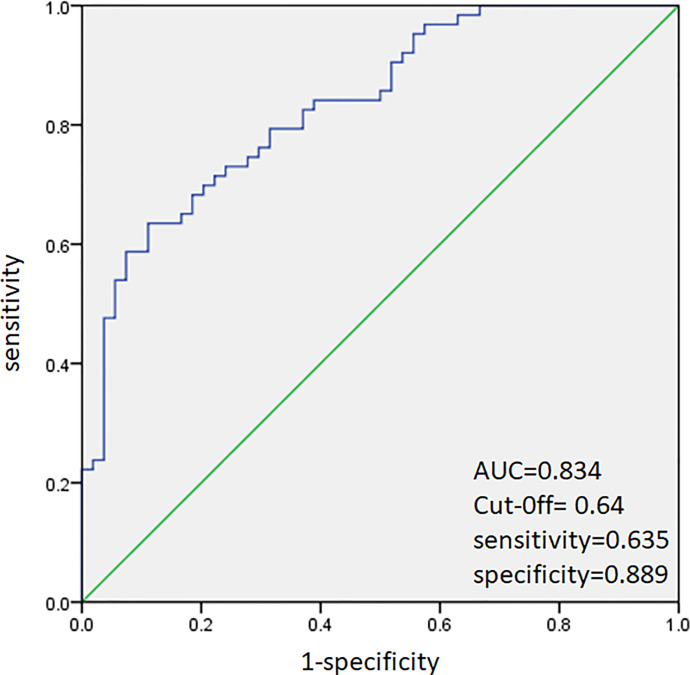
Receiver operating characteristic curve analysis for prediction of central lymph node metastases using the multivariate model in HT patients.

**Figure 3:**
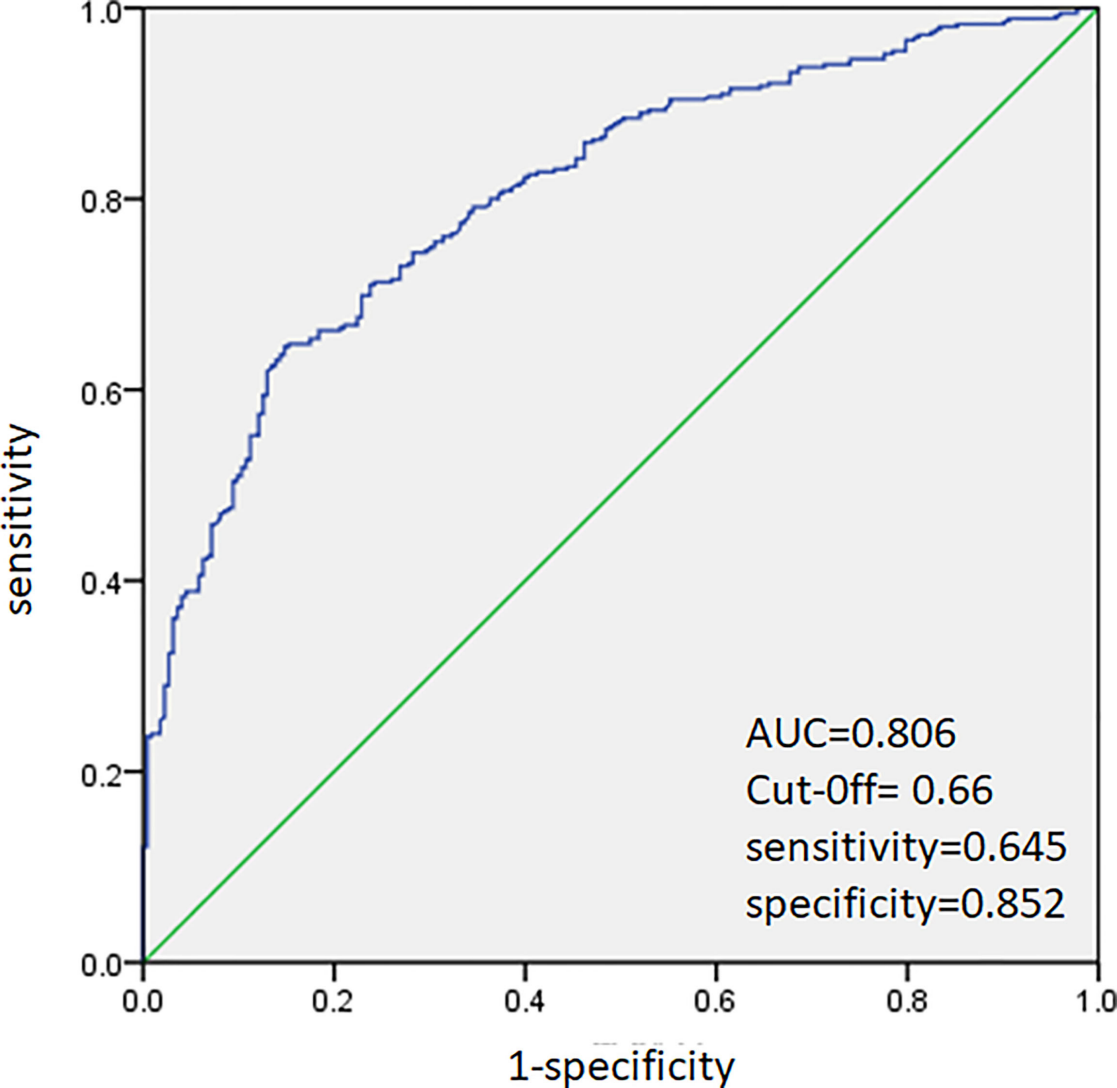
Receiver operating characteristic curve analysis for prediction of central lymph node metastases using the multivariate model in non-HT patients.

ROC analysis was performed to predict CLNM in HT and non-HT patients, respectively. The AUC was 83.4% (**Figure 2**) in HT patients. A cutoff point for prediction of CLNM in HT group was defined as a value 64%. The sensitivity of the multivariate model was 63.5%, and specificity was 88.9% for prediction CLNM in HT patients (**Figure 2**). For non-HT patients, the AUC was 80.6%. A cutoff point for prediction of CLNM was defined as a value 66%; the sensitivity of the multivariate model was 64.5%, and specificity was 85.2% (**Figure 3**).

## Discussion

HT, which was first identified by Hashimoto in 1912, is regarded as a destructive autoimmune disease of thyroid (24). An obvious increase in the incidence of the coexistence of PTC and HT has been reported during the past 20 years, and the impact of HT in PTC has been a research hotspot (25–27). Most of the studies show that PTC in the presence of HT is associated with an earlier stage of disease at the time of initial treatment and a better prognosis (8–11, 28, 29). However, only a handful of studies investigate the influence of HT on lymph node metastasis in PTC. Our study confirmed the influence of HT on the clinical characteristics of PTC and also showed that the predictive factors of CLNM varied in HT and non-HT group.

Our results showed that there were more female patients in HT group than in non-HT group. Our study also indicated that the TSH in HT patients was higher than that in patients with PTC alone. These results were consistent with many other previous observations (24, 30). HT, which is characterized by the infiltration of abundant lymphocytes, fibrosis, and parenchymal atrophy of thyroid tissue, is an autoimmune inflammation of the thyroid. The autoimmune response of HT can result in an increase in TSH (31, 32). What’s more, the incidence of PTC increases gradually with the increase in TSH levels. Thus, HT patients with thyroid nodules need to be carefully monitored in our clinical practice.

Some studies have shown that the rate of CLNM is lower in patients with PTC coexistent with HT (33, 34). However, there was no statistical significance in the rate of CLNM between the HT group and non-HT group in this study. We had a very interesting finding that although the number of lymph node dissections in the central compartment region of PTC coexistent with the HT group were more than those in the PTC group, the number of metastatic lymph nodes were fewer in the HT group. Similar findings have also been found in previous studies (27, 35, 36). In fact, it is unclear whether HT influences the CLNM in PTC patients due to lack of relevant studies. Increasing results suggest that the inflammatory process of thyroiditis confers a protective effect on PTC.

In fact, many patients with PTC coexistent with HT always have more enlarged lymph nodes in the neck (37), and it brings more difficulties in determining the metastasis of lymph node using preoperative color Doppler ultrasound. PTC patients with HT appear to undergo a more excessive lymph node dissection because of the presence of more enlarged lymphadenopathy identified at the time of thyroidectomy, which is likely to result in more postoperative complications. The rates of RLN injury have been reported to increase significantly during therapeutic central compartment lymph node dissection (38). Previous systematic meta-analyses have reported RLN injury rates of 2.8%–9.8% (39). Voice, swallowing, and coughing can be affected in RLN injury. The voice is weak and breathy with a loss of vocal projection and phonation time, which may have greater impact on patients working in teaching, training, management, and administrative support and make them more vulnerable to experiencing voice disorders in their daily lives. PTC patients with HT are more likely to have enlarged lymph nodes, thus prompting the surgeon to perform a more extensive CND. However, an overly aggressive dissection that may damage the normal function of the RLN is not necessary (37). Therefore, the accurate identification of risk factors of CLNM is helpful to evaluate the status of CLN and determine the necessity of lymph node dissection, especially for PTC patients with HT (30).

Previous studies have revealed that age of onset and multifocality are independently associated with CLNM in PTC (34, 40). It has been established that the risk of CLNM positively correlates with the number of tumor foci (34, 41), and tumor size >1 cm was also reported to be a risk factor of CLNM in PTC (42, 43). Our study confirmed that age, multifocality, and tumor size were independent risk factors of CLNM in PTC patients. In addition, male gender, irregular nodules, and presence of suspicious CLN on US were independent risk factors of CLNM.

We further explored differences in CLNM between patients with and without HT. Our study confirmed the predictive value of tumor size and presence of suspicious CLN on US in both groups with and without HT and showed that age, gender, and the number of suspicious CLN on US lost their predictive value for CLNM in PTC patients with HT. It suggested that HT might have an impact against aggressive behaviors and play a protective role in the natural history to some extent.

Then, we performed different multivariate models to calculate the probability of CLNM for HT and non-HT patients, respectively. ROC analysis was performed to predict CLNM in both groups (AUCs, 83.4% and 80.6%, respectively). Compared with the whole PTC group, the sensitivity of those models in predicting CLNM was both higher for HT and non-HT groups (63.5% *versus* 61% and 64.5% *versus* 61%, respectively), and the specificity of those models was a little higher for HT group (88.9% versus 86%), while the specificity of those models was a little lower for non-HT group than the whole PTC group (85.2% versus 86%).

However, the present study also had some potential limitations. A limitation of the present study is its retrospective nature. Further prospective studies are needed to clarify the potential causal relationship between HT and PTC. Second, our study is a single-center study, and the sample size is a little small. Further multicenter studies with a larger number of patients are needed. What is worse, there are not enough statistical data and tracking data for postoperative complications. Therefore, multicenter collaboration and long-term follow-up are needed to obtain more reliable results in the future.

## Conclusions

In summary, our study explored and confirmed the impact of HT on the predictive risk factors of CLNM in PTC. PTC combined with HT is more common in women, and TSH level in HT group is higher than that in patients with PTC alone. Regardless of the fact that HT is not a related risk factor of CLNM in PTC, our result suggested that different predictive systems should be used for HT and non-HT patients, respectively, to have a more accurate evaluation of CLNM in clinics. Considering the possible influence of HT on CLNM in PTC, the scope of lymph node dissection should be performed more accurately in HT patients.

## Data Availability Statement

The raw data supporting the conclusions of this article will be made available by the authors, without undue reservation.

## Ethics Statement

The studies involving human participants were reviewed and approved by the institutional review board of the First Affiliated Hospital of Xi’an Jiaotong University. Written informed consent to participate in this study was provided by the participants’ legal guardian/next of kin.

## Author Contributions

YS designed the research. YL, HL, SZ, BS, and YS conducted research. HL and SZ the analyzed data. YL wrote the initial draft of the manuscript. BS and YS revised the manuscript. All authors contributed to the article and approved the submitted version.

## Funding

This study was supported by the Shaanxi Provincial Natural Science Foundation (Grant No. 2021SF-010) and the Basic Research Foundation of the First Affiliated Hospital of Xi’an Jiaotong University (Grant No. 2020QN-24).

## Conflict of Interest

The authors declare that the research was conducted in the absence of any commercial or financial relationships that could be construed as a potential conflict of interest.

## Publisher’s Note

All claims expressed in this article are solely those of the authors and do not necessarily represent those of their affiliated organizations, or those of the publisher, the editors and the reviewers. Any product that may be evaluated in this article, or claim that may be made by its manufacturer, is not guaranteed or endorsed by the publisher.

## References

[1] Ahmed R Al-Shaikh S Akhtar M. Hashimoto Thyroiditis: A Century Later. Adv Anat Pathol (2012) 19:181–6. doi: 10.1097/PAP.0b013e3182534868 22498583

[2] Latina A Gullo D Trimarchi F Benvenga S. Hashimoto's Thyroiditis: Similar and Dissimilar Characteristics in Neighboring Areas. Possible Implications for the Epidemiology of Thyroid Cancer. PloS One (2013) 8:e55450. doi: 10.1371/journal.pone.0055450 23526929 PMC3601092

[3] Siegel RL Miller KD Jemal A. Cancer Statistics, 2015. CA Cancer J Clin (2015) 65:5–29. doi: 10.3322/caac.21254 25559415

[4] Mao Y Xing M. Recent Incidences and Differential Trends of Thyroid Cancer in the USA. Endocr Relat Cancer (2016) 23:313–22. doi: 10.1530/ERC-15-0445 PMC4891202 26917552

[5] Lindsay S Dailey ME Friedlander J Yee G Soley MH. Chronic Thyroiditis: A Clinical and Pathologic Study of 354 Patients. J Clin Endocrinol Metab (1952) 12:1578–600. doi: 10.1210/jcem-12-12-1578 13022745

[6] Lee JH Kim Y Choi JW Kim YS. The Association Between Papillary Thyroid Carcinoma and Histologically Proven Hashimoto's Thyroiditis: A Meta-Analysis. Eur J Endocrinol (2013) 168:343–9. doi: 10.1530/EJE-12-0903 23211578

[7] Schaffler A Palitzsch KD Seiffarth C Hohne HM Riedhammer FJ Hofstadter F. Coexistent Thyroiditis Is Associated With Lower Tumour Stage in Thyroid Carcinoma. Eur J Clin Invest (1998) 28:838–44. doi: 10.1046/j.1365-2362.1998.00363.x 9792998

[8] Matsubayashi S Kawai K Matsumoto Y Mukuta T Morita T Hirai K. The Correlation Between Papillary Thyroid Carcinoma and Lymphocytic Infiltration in the Thyroid Gland. J Clin Endocrinol Metab (1995) 80:3421–4. doi: 10.1210/jcem.80.12.8530576 8530576

[9] Kashima K Yokoyama S Noguchi S Murakami N Yamashita H Watanabe S. Chronic Thyroiditis as a Favorable Prognostic Factor in Papillary Thyroid Carcinoma. Thyroid (1998) 8:197–202. doi: 10.1089/thy.1998.8.197 9545105

[10] Loh KC Greenspan FS Dong F Miller TR Yeo PP. Influence of Lymphocytic Thyroiditis on the Prognostic Outcome of Patients With Papillary Thyroid Carcinoma. J Clin Endocrinol Metab (1999) 84:458–63. doi: 10.1210/jcem.84.2.5443 10022401

[11] Singh B Shaha AR Trivedi H Carew JF Poluri A Shah JP. Coexistent Hashimoto's Thyroiditis With Papillary Thyroid Carcinoma: Impact on Presentation, Management, and Outcome. Surgery (1999) 126:1070–6; discussion 6-7. doi: 10.1067/msy.2099.101431 10598190

[12] Del Rio P Cataldo S Sommaruga L Concione L Arcuri MF Sianesi M. The Association Between Papillary Carcinoma and Chronic Lymphocytic Thyroiditis: Does It Modify the Prognosis of Cancer? Minerva Endocrinol (2008) 33:1–5. doi: 10.1159/000127404 18277374

[13] Hughes CJ Shaha AR Shah JP Loree TR. Impact of Lymph Node Metastasis in Differentiated Carcinoma of the Thyroid: A Matched-Pair Analysis. Head Neck (1996) 18:127–32. doi: 10.1002/(SICI)1097-0347(199603/04)18:2<127::AID-HED3>3.0.CO;2-3 8647677

[14] Ito Y Tomoda C Uruno T Takamura Y Miya A Kobayashi K. Clinical Significance of Metastasis to the Central Compartment From Papillary Microcarcinoma of the Thyroid. World J Surg (2006) 30:91–9. doi: 10.1007/s00268-005-0113-y 16369721

[15] Lee DW Ji YB Sung ES Park JS Lee YJ Park DW. Roles of Ultrasonography and Computed Tomography in the Surgical Management of Cervical Lymph Node Metastases in Papillary Thyroid Carcinoma. Eur J Surg Oncol (2013) 39:191–6. doi: 10.1016/j.ejso.2012.07.119 22863305

[16] Ahn JE Lee JH Yi JS Shong YK Hong SJ Lee DH. Diagnostic Accuracy of CT and Ultrasonography for Evaluating Metastatic Cervical Lymph Nodes in Patients With Thyroid Cancer. World J Surg (2008) 32:1552–8. doi: 10.1007/s00268-008-9588-7 18408961

[17] Morita S Mizoguchi K Suzuki M Iizuka K. The Accuracy of (18)[F]-Fluoro-2-Deoxy-D-Glucose-Positron Emission Tomography/Computed Tomography, Ultrasonography, and Enhanced Computed Tomography Alone in the Preoperative Diagnosis of Cervical Lymph Node Metastasis in Patients With Papillary Thyroid Carcinoma. World J Surg (2010) 34:2564–9. doi: 10.1007/s00268-010-0733-8 20645089

[18] Hwang HS Orloff LA. Efficacy of Preoperative Neck Ultrasound in the Detection of Cervical Lymph Node Metastasis From Thyroid Cancer. Laryngoscope (2011) 121:487–91. doi: 10.1002/lary.21227 21344423

[19] Lynch J Parameswaran R. Management of Unilateral Recurrent Laryngeal Nerve Injury After Thyroid Surgery: A Review. Head Neck (2017) 39:1470–8. doi: 10.1002/hed.24772 28370683

[20] Beka E Gimm O. Voice Changes Without Laryngeal Nerve Alterations After Thyroidectomy: The Need For Prospective Trials - A Review Study. J Voice (2021). doi: 10.1016/j.jvoice.2021.07.012 34404582

[21] Pereira JA Girvent M Sancho JJ Parada C Sitges-Serra A. Prevalence of Long-Term Upper Aerodigestive Symptoms After Uncomplicated Bilateral Thyroidectomy. Surgery (2003) 133:318–22. doi: 10.1067/msy.2003.58 12660645

[22] Sun Y Lv H Zhang S Bai Y Shi B. Gender-Specific Risk of Central Compartment Lymph Node Metastasis in Papillary Thyroid Carcinoma. Int J Endocrinol (2018) 2018:6710326. doi: 10.1155/2018/6710326 29713344 PMC5866883

[23] Amin MB Edge SB Greene FL Byrd DR Brookland RK Washington MK. AJCC Cancer Staging Manual Eighth Edition. Switzerland: Springer International Publishing (2017) p. 873–90.

[24] Liang J Zeng W Fang F Yu T Zhao Y Fan X. Clinical Analysis of Hashimoto Thyroiditis Coexistent With Papillary Thyroid Cancer in 1392 Patients. Acta Otorhinolaryngol Ital (2017) 37:393–400. doi: 10.14639/0392-100X-1709 29165434 PMC5720867

[25] Tamimi DM. The Association Between Chronic Lymphocytic Thyroiditis and Thyroid Tumors. Int J Surg Pathol (2002) 10:141–6. doi: 10.1177/106689690201000207 12075407

[26] Kebebew E Treseler PA Ituarte PH Clark OH. Coexisting Chronic Lymphocytic Thyroiditis and Papillary Thyroid Cancer Revisited. World J Surg (2001) 25:632–7. doi: 10.1007/s002680020165 11369991

[27] Zhang L Li H Ji QH Zhu YX Wang ZY Wang Y. The Clinical Features of Papillary Thyroid Cancer in Hashimoto's Thyroiditis Patients From an Area With a High Prevalence of Hashimoto's Disease. BMC Cancer (2012) 12:610. doi: 10.1186/1471-2407-12-610 23256514 PMC3547693

[28] Kim EY Kim WG Kim WB Kim TY Kim JM Ryu JS. Coexistence of Chronic Lymphocytic Thyroiditis Is Associated With Lower Recurrence Rates in Patients With Papillary Thyroid Carcinoma. Clin Endocrinol (Oxf) (2009) 71:581–6. doi: 10.1111/j.1365-2265.2009.03537.x 19222495

[29] Yoon YH Kim HJ Lee JW Kim JM Koo BS. The Clinicopathologic Differences in Papillary Thyroid Carcinoma With or Without Co-Existing Chronic Lymphocytic Thyroiditis. Eur Arch Otorhinolaryngol (2012) 269:1013–7. doi: 10.1007/s00405-011-1732-6 21822854

[30] Jin K Li L Liu Y Wang X. The Characteristics and Risk Factors of Central Compartment Lymph Node Metastasis in Cn0 Papillary Thyroid Carcinoma Coexistent With Hashimoto's Thyroiditis. Gland Surg (2020) 9:2026–34. doi: 10.21037/gs-20-699 PMC7804536 33447553

[31] Liu X Zhu L Cui D Wang Z Chen H Duan Y. Coexistence of Histologically Confirmed Hashimoto's Thyroiditis With Different Stages of Papillary Thyroid Carcinoma in a Consecutive Chinese Cohort. Int J Endocrinol (2014) 2014:769294. doi: 10.1155/2014/769294 25505911 PMC4255062

[32] Resende de Paiva C Gronhoj C Feldt-Rasmussen U von Buchwald C. Association Between Hashimoto's Thyroiditis and Thyroid Cancer in 64,628 Patients. Front Oncol (2017) 7:53. doi: 10.3389/fonc.2017.00053 28443243 PMC5385456

[33] Wen X Wang B Jin Q Zhang W Qiu M. Thyroid Antibody Status is Associated With Central Lymph Node Metastases in Papillary Thyroid Carcinoma Patients With Hashimoto's Thyroiditis. Ann Surg Oncol (2019) 26:1751–8. doi: 10.1245/s10434-019-07256-4 30937662

[34] Zhu F Shen YB Li FQ Fang Y Hu L Wu YJ. The Effects of Hashimoto Thyroiditis on Lymph Node Metastases in Unifocal and Multifocal Papillary Thyroid Carcinoma: A Retrospective Chinese Cohort Study. Med (Baltimore) (2016) 95:e2674. doi: 10.1097/MD.0000000000002674 PMC4753890 26871795

[35] Jara SM Carson KA Pai SI Agrawal N Richmon JD Prescott JD. The Relationship Between Chronic Lymphocytic Thyroiditis and Central Neck Lymph Node Metastasis in North American Patients With Papillary Thyroid Carcinoma. Surgery (2013) 154:1272–80; discussion 80-2. doi: 10.1016/j.surg.2013.07.021 24238047

[36] Schneider DF Chen H Sippel RS. Impact of Lymph Node Ratio on Survival in Papillary Thyroid Cancer. Ann Surg Oncol (2013) 20:1906–11. doi: 10.1245/s10434-012-2802-8 PMC3609925 23263904

[37] Lai V Yen TW Rose BT Fareau GG Misustin SM Evans DB. The Effect of Thyroiditis on the Yield of Central Compartment Lymph Nodes in Patients With Papillary Thyroid Cancer. Ann Surg Oncol (2015) 22:4181–6. doi: 10.1245/s10434-015-4551-y 25851341

[38] Roh JL Kim JM Park CI. Central Compartment Reoperation for Recurrent/Persistent Differentiated Thyroid Cancer: Patterns of Recurrence, Morbidity, and Prediction of Postoperative Hypocalcemia. Ann Surg Oncol (2011) 18:1312–8. doi: 10.1245/s10434-010-1470-9 21140230

[39] Cirocchi R Arezzo A D'Andrea V Abraha I Popivanov GI Avenia N. Intraoperative Neuromonitoring Versus Visual Nerve Identification for Prevention of Recurrent Laryngeal Nerve Injury in Adults Undergoing Thyroid Surgery. Cochrane Database Syst Rev (2019) 1:CD012483. doi: 10.1002/14651858.CD012483.pub2 30659577 PMC6353246

[40] Lee YS Lim YS Lee JC Wang SG Son SM Kim SS. Ultrasonographic Findings Relating to Lymph Node Metastasis in Single Micropapillary Thyroid Cancer. World J Surg Oncol (2014) 12:273. doi: 10.1186/1477-7819-12-273 25169012 PMC4159533

[41] Siddiqui S White MG Antic T Grogan RH Angelos P Kaplan EL. Clinical and Pathologic Predictors of Lymph Node Metastasis and Recurrence in Papillary Thyroid Microcarcinoma. Thyroid (2016) 26:807–15. doi: 10.1089/thy.2015.0429 27117842

[42] Park JP Roh JL Lee JH Baek JH Gong G Cho KJ. Risk Factors for Central Neck Lymph Node Metastasis of Clinically Noninvasive, Node-Negative Papillary Thyroid Microcarcinoma. Am J Surg (2014) 208:412–8. doi: 10.1016/j.amjsurg.2013.10.032 24602323

[43] Konturek A Barczynski M Nowak W Wierzchowski W. Risk of Lymph Node Metastases in Multifocal Papillary Thyroid Cancer Associated With Hashimoto's Thyroiditis. Langenbecks Arch Surg (2014) 399:229–36. doi: 10.1007/s00423-013-1158-2 PMC3916705 24407910

