# Supplementary material for: The Impact of Coexistent Hashimoto’s Thyroiditis on Central Compartment Lymph Node Metastasis in Papillary Thyroid Carcinoma
Source: Front Endocrinol (Lausanne). 2021 Nov 16;12:772071. doi: 10.3389/fendo.2021.772071 (PMC8635140; doi:10.3389/fendo.2021.772071)
Supplement: Supplementary file 1 [file Table_1.doc]

**TABLE S1 |**. Sensitivity and specificity of ultrasonography for the detection of suspicious lymph nodes in PTC.

|  | HT | | non-HT | |
| --- | --- | --- | --- | --- |
|  | CLNM | NCLNM | CLNM | NCLNM |
| presence of suspicious CLN on US | 21 | 2 | 139 | 13 |
| absence of suspicious CLN on US | 58 | 49 | 246 | 228 |

**TABLE S2** **|** Multivariate analysis of the correlation between clinical factors of the primary tumor and rate of CLNM in HT and non-HT patients of stage cN0.

| **Variables** | **OR** | **CI** | | **P** |
| --- | --- | --- | --- | --- |
| ***non-HT group*** | | | | |
| Gender（male） | 1.990 | （1.224-3.236） | 0.006 | |
| Age (≤45 y) | 3.205 | （2.115—4.856） | ＜0.001 | |
| Margin (regular) | 0.652 | （0.431-0.988） | 0.044 | |
| Tumor size (＞10mm) | 2.186 | （1.363-3.505） | 0.001 | |
| ***HT group*** | | | | |
| Age (≤45 y) | 2.991 | （1.031-8.675） | 0.044 | |
| Tumor size (＞10mm) | 4.679 | （1.446-15.136） | 0.010 | |
